# Mortality and life table analysis in a young cohort of pet cats in the UK

**DOI:** 10.1177/1098612X251314689

**Published:** 2025-04-11

**Authors:** Aimee R Taylor, Jennifer McDonald, Rae Foreman-Worsley, Angie Hibbert, Emily J Blackwell

**Affiliations:** 1 Bristol Veterinary School, University of Bristol, Bristol, UK; 2 Feline Welfare Directorate, Cats Protection, Haywards Heath, UK; 3 The Feline Centre, Langford Vets, Langford, UK

**Keywords:** Bristol Cats study, cause of death, mortality, longevity, survival, life table

## Abstract

**Objectives:**

Mortality data represent an important resource for understanding population health that previously have mostly been extrapolated from veterinary records. The aims of this study were to explore mortality for a cohort of pet cats in the UK using data from owner-completed questionnaires, thus potentially representing a more comprehensive population, and to determine the all-cause mortality and survival probabilities.

**Methods:**

Data were collated from the ‘Bristol Cats’ study questionnaires, owner communications and medical records for the cohort’s first 8 years of life. Mortality was classified by organ system, disease or event, and analysed by age and life stage. Age-specific mortality and cumulative survival probability curves were constructed using life table analysis.

**Results:**

Of 2444 cats recruited into the ‘Bristol Cats’ study, at least 362 died before reaching the age of 9 years. The most common cause of death in cats up to the age of 8 years was road traffic accident (RTA; 45.6%). For kittens (aged <1 year), the most common causes were RTA (61.2%), feline infectious peritonitis (11.9%) and other trauma (7.5%). The most common causes of mortality in young adult cats (aged 1–6 years) were RTA (49.6%), non-specified (14.4%), renal disease (6.5%) and cardiovascular disease (6.5%). Cats aged up to 2 years had an annual probability of dying in the range of 2.8–3.1%, which decreased between 2 and 3 years of age to 1.7%. Thereafter, the probability of dying gradually increased with age, with the oldest age group (aged 7–8 years) having a 3.6% probability of dying.

**Conclusions and relevance:**

This study identified important differences in causes of mortality for the first two life stages in a population of cats that may not be fully accounted for in medical records. Life table analysis documented an increasing probability of death with age after year interval 2–3 with a higher mortality risk for cats aged up to 2 years.

## Introduction

There are an estimated 11 million pet cats in the UK, with 26% of households owning one or more cats.^1^ However, there is a paucity of information in the veterinary literature regarding their mortality and survival rates, despite this type of information being an important resource that can be utilised to improve population health, welfare and longevity. Most of the published mortality studies are based on surveillance data and information obtained from medical^2–5^ or insurance records.^6^ Cohort life table analysis is a useful method of analysing long-term mortality trends and life expectancy; this method evaluates demographic patterns and the impact of risk factors over time within specific groups. Worldwide, there are only three previous life table reports for pet cats based on veterinary surveillance data (UK),^5^ clinical records (USA)^7^ and cemetery records (Japan).^8^ Surveillance studies rely on owners presenting their pets to a veterinary practice or submitting an insurance claim to capture the incidence of illness or death. In the UK, at the time of writing, approximately 10% of pet cats were not registered with a veterinary clinic, only 61% received regular booster vaccinations and 48% were insured.^1^ There may be various socioeconomic, geographical or personal reasons why some pet owners choose not to use veterinary services or insure their pets; however, as a result, there is a subset of the pet cat population unaccounted for via medical records or insurance data.

The ‘Bristol Cats’ study (https://www.bristol.ac.uk/vet-school/research/projects/cats/) is an ongoing, unique, longitudinal cohort study, designed to improve knowledge of common feline health, behavioural and welfare problems. Kittens and their owners were recruited between 2010 and 2013. The study was advertised using a variety of methods (eg, via pet forums, cat interest websites, social media and posters in pet shops, rescue centres and veterinary practices) to minimise recruitment selection bias.^9^ An annual prize draw for a supermarket voucher is provided as incentivisation. Data are collected primarily prospectively via questionnaires, with corroborating or additional information obtained retrospectively from the cats’ medical records. The questionnaires capture information about various aspects of the cats’ lives, including their environment, lifestyle, health and behaviour, regardless of their attendance at veterinary clinics. Mortality data from ‘Bristol Cats’ were first assessed when the cats were aged 12 months and this analysis found that 71.4% of the cats that were known to have been involved in an RTA experienced fatal injuries.^10^ The aims of the current study were to reassess mortality for the ‘Bristol Cats’ cohort by age and life stage, and to create life tables now that they are older.

## Materials and methods

### Data collection

Data for the project were collected as part of the ‘Bristol Cats’ study, which has ethical approval from the University of Bristol (reference number UIN/13/026). Owners were asked to complete questionnaires at recruitment, when their cats were aged 8–16 weeks (questionnaire 1 [Q1]), 6 months (Q2), 12 months (Q3), 1.5 years (Q4), 2.5 years (Q5), 4 years (Q6) and annually thereafter. As ‘Bristol Cats’ is an ongoing cohort study with cats not yet in their senior years, for the present study data were collated from the most recent complete data set – Q1–Q10 – relating to the first 8 years of the cats’ lives. Deceased cats were those that were reported by their owners to have died, either via subsequent questionnaires or in direct communications with the ‘Bristol Cats’ team. Causes of mortality were categorised by organ system, pathophysiology or event, based primarily on the questionnaire information provided by the owners. The categories used were guided by a previous study^2^ (Table 1). Where the owner’s recollection was unclear or specifically stated terms such as ‘suspected’ or ‘likely’, the veterinary medical records were obtained for review by the first author (ART). Mortality cause was categorised according to veterinary interpretation; owing to ambiguity in four cases, confirmation was sought from a feline medicine specialist (AH). Where the owner had not specified a clear cause and medical records were not available for review, the cause of death was classified as non-specified.

**Table 1:** Categorisation of causes of death reported by pet cat owners and in medical records, including examples of terminology used in the ‘Bristol Cats’ questionnaires

Cause of death	Example(s) of details from questionnaires and medical records
RTA	Found on the road, hit by a car/van
Other trauma	Dog attack, shot, strangled
FIP	Died of FIP, PTS because of FIP, contracted FIP
Neoplasia	Cancer, cancerous tumour or any specific neoplasm name
Cardiovascular disease	Congestive heart failure, aortic thromboembolism, hypertrophic cardiomyopathy, dilated cardiomyopathy or enlarged heart; heart attack (with supportive history of cardiac disease)
Respiratory disease	Aspiration pneumonia, pyothorax, *Bordetella* bronchopneumonia
Gastrointestinal disease	Vomiting, diarrhoea, pancreatic disease, pancreatitis
Hepatic disease	Liver disease
Renal disease	Kidney/renal failure, chronic kidney disease, suspected ethylene glycol toxicity, suspected lily toxicity, polycystic kidney disease, nephrolithiasis
Urinary disorder	Blocked bladder, bladder rupture
Endocrine disorder	Diabetes mellitus
Haematological disorder	IMHA
Neurological disorder	Seizures, lesions on the brain, fits, degenerative brain disorder, brain damage from inner ear infection, brain tumour
Behavioural condition	‘Behaviour problems’
Surgical complication	Peri- and postoperative (all involved neutering)
Toxicity	Poisoned (not including suspected ethylene glycol or lily toxicity)
Sudden death	Heart attack (without supportive history of cardiac disease), found deceased in bed/on driveway/in garden (with no history of illness or evidence of trauma)
Non-specified	Died/PTS (with no additional information available)

FIP = feline infectious peritonitis; IMHA = immune-mediated haemolytic anaemia; PTS = put to sleep; RTA = road traffic accident

The date of death was either reported by the owners, obtained from the medical records or listed as not available. The age at death was preferentially calculated from the birthdate and date of death or explicitly recorded by the owner. For cases where this was not possible, age was approximated based on the questionnaire in which the death was reported. Between annual questionnaires, this yielded an approximate age accurate to the nearest year, as all cats were enrolled at 8–16 weeks old. However, because Q4, Q5 and Q6 were completed when the cats were aged 1.5 years, 2.5 years and 4 years, respectively, for any deaths reported in these questionnaires (without a known birthdate and date of death) it was not possible to approximate the age of death accurately to the nearest year. Any cat that died before the age of 12 months was recorded as being ‘0’ years old. Recorded demographic data included sex, neuter status and pedigree status.

### Statistical analysis

Descriptive statistics were used to summarise the causes of mortality for all the cats in the study and then by life stage (kitten, aged <1 year, and young adult, aged 1–6 years) as defined by the American Association of Feline Practitioners.^11^ For the life table analysis, cats without a recorded date of birth (n = 245), approximate age of death accurate to the nearest year (n = 4) or with incomplete data (n = 5) were excluded. Consequently, 2190 cats were included in the life table analysis. Birthdate and date of most recent questionnaire completion were used to assess the individual’s ongoing participation at specific age time points; at each age time point within the analysis, cats were categorised as either remaining in the study, a dropout or deceased. Dropouts were defined as cats that were not reported to have died, but whose owners had not completed the most recent questionnaires. To minimise the risk of misclassifying a cat as a dropout, when owners may decide to rejoin the study at a later date, we excluded the two most recent questionnaires (Q9 and Q10) from the analysis, but considered their completion in the classification of dropouts, therefore requiring owners to have missed a minimum of two consecutive recent questionnaires to be classified as a dropout. The life tables generated from this information provided age-specific survival and mortality rates for the population. Mortality at age x (q_x_) was calculated as the number of deaths in an interval divided by the number of cats participating at the start of the interval, with the probability of surviving to a certain age (l_x_) calculated by multiplying the annual survival probability (1 – q_x_) at each age interval from birth to that age. Excel version 2408 (Microsoft) was used to conduct the data cleaning and life table analysis.

## Results

### All-cause mortality

Of the 2444 cats recruited into the ‘Bristol Cats’ study, at least 362 died of various causes before reaching 9 years old (between recruitment and September 2022). Of these, birthdates were available for 334 cats and date of death information was available for 219 cats. Using this information, the exact age at death was calculated for 201 cats. Owner recollection of the cat’s age at death was used for 35 cases and an approximation of the age based on questionnaire number was used for 120. Of the deceased cases, 55.5% were male (n = 201) and 44.5% were female (n = 161). Neuter status was available for 79.8% (n = 289); 47.1% were male neutered, 36.7% were female neutered, 9.0% were female entire and 7.3% were male entire. Data regarding pedigree status were available for 97.8% of cases, of which 26.1% were pedigree (n = 91) and 73.9% were non-pedigree (n = 257). The cause of death was reported by the owner for 351 cases; for 11 cases, this information was obtained from the cat’s medical records. The five most commonly attributed causes of mortality for all the cats were road traffic accident (RTA) (n = 165, 45.6%), non-specified (n = 48, 13.3%), renal disease (n = 24, 6.6%), cardiovascular disease (n = 23, 6.4%) and neoplasia (n = 22, 6.1%) (Table 2). For kittens (aged <1 year), the most common causes were RTA (n = 41, 61.2%), feline infectious peritonitis (FIP) (n = 8, 11.9%%), other trauma (n = 5, 7.5%%), surgical complications (n = 4, 6.0%) and toxin ingestion (n = 2, 3.0%) (Table 2). The most common causes of mortality for young adult cats (aged 1–6 years) were RTA (n = 114, 49.6%), non-specified (n = 33, 14.4%%), renal disease (n = 15, 6.5%), cardiovascular disease (n = 15, 6.5%) and sudden death (n = 11, 4.8%) (Table 2).

**Table 2:** Causes of mortality for pet cats up to 8 years of age (n = 362), 1–6 years of age (n = 230) and <1 year of age (n = 67) in the ‘Bristol Cats’ study as reported by their owners and in medical records

Cause of death	Up to 8 years old (n = 362)		1–6 years old (n = 230)		<1 year old (n = 67)	
	Rank	Deaths	Rank	Deaths	Rank	Deaths
RTA	1	165 (45.58)	1	114 (49.57)	1	41 (61.19)
Non-specified	2	48 (13.26)	2	33 (14.35)	6	1 (1.49)
Renal disease	3	24 (6.63)	3	15 (6.52)	–	0 (0.00)
Cardiovascular disease	4	23 (6.35)	3	15 (6.52)	6	1 (1.49)
Neoplasia	5	22 (6.08)	5	10 (4.35)	–	0 (0.00)
Sudden death	6	17 (4.70)	4	11 (4.78)	6	1 (1.49)
FIP	7	14 (3.87)	8	5 (2.17)	2	8 (11.94)
Other trauma	7	14 (3.87)	6	8 (3.48)	3	5 (7.46)
Neurological disorder	8	10 (2.76)	7	7 (3.04)	6	1 (1.49)
Surgical complication	9	5 (1.38)	–	0 (0.00)	4	4 (5.97)
Hepatic disease	10	4 (1.10)	9	3 (1.30)	–	0 (0.00)
Respiratory disease	10	4 (1.10)	9	3 (1.30)	6	1 (1.49)
Gastrointestinal disease	11	3 (0.83)	10	2 (0.87)	6	1 (1.49)
Toxin	11	3 (0.83)	11	1 (0.43)	5	2 (2.99)
IMHA	12	2 (0.55)	–	0 (0.00)	6	1 (1.49)
Urinary disorder	12	2 (0.55)	11	1 (0.43)	–	0 (0.00)
Behavioural condition	13	1 (0.28)	11	1 (0.43)	–	0 (0.00)
Endocrine disorder	13	1 (0.28)	11	1 (0.43)	–	0 (0.00)

Data are n (%)

FIP = feline infectious peritonitis; IMHA = immune-mediated haemolytic anaemia; RTA = road traffic accident

### Life table analysis

**Figure 1:**
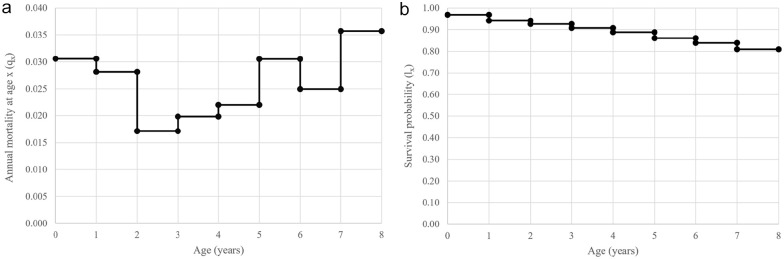
(a) Age-specific mortality (q_x_) curve and (b) age-specific survival (l_x_) curve obtained from life table analysis of the ‘Bristol Cats’ cohort

Of the 2190 individual cats that were incorporated in the life table analysis, by the age of 8 years, 49% (n = 1071) were classed as dropouts and 13% (n = 294) were confirmed as deceased; the difference in total number of deceased cats compared with the all-cause mortality data set reflects those cases that were excluded. Demographic data are available in Table 3. Life table analysis, accounting for dropouts, found that cats aged up to 2 years had an annual probability of dying in the range of 2.8–3.1%, which decreased at 2–3 years of age to 1.7%. Thereafter, mortality rates gradually increased, with a 3.6% probability of cats dying at 7–8 years of age (Figure 1a). Overall, 81% of cats survived until the age of 8 years (Figure 1b).

**Table 3:** Demographic data for cats included in the life table analysis (n = 2190) that had died (n = 294), dropped out (n = 1071) or were still alive (n = 825) in the ‘Bristol Cats’ study

Demographic variables	Deceased (n = 294)	Dropped out (n = 1071)	Still alive (n = 825)
Sex/neuter status			
Male neutered	137 (46.6)	439 (41.0)	432 (52.4)
Female neutered	108 (36.7)	380 (35.5)	382 (46.3)
Male entire	22 (7.5)	95 (8.9)	3 (0.4)
Female entire	26 (8.8)	135 (12.6)	6 (0.7)
Unknown	1 (0.3)	22 (2.1)	2 (0.2)
Pedigree status			
Non-pedigree	206 (70.0)	789 (73.7)	642 (77.8)
Pedigree	78 (26.5)	234 (21.8)	168 (20.4)
Unknown	10 (3.4)	48 (4.5)	15 (1.8)

Data are n (%)

## Discussion

This is the first UK-wide study to use data from a prospective cohort study to assess all-cause mortality and survival rates in pet cats. Unlike most other mortality studies, data are included from the subset of cats in the general population that do not attend veterinary clinics or have insurance, and therefore potentially represents a more comprehensive UK pet population; 21.5% of the ‘Bristol Cats’ cohort that completed Q10 (age 8 years) had not visited a veterinarian in the preceding year.

Trauma, namely RTA, is well documented as a common cause of feline mortality,^2,3,12^ especially in the UK, where allowing cats outdoor access is common. In this cohort of cats, RTA remained the most common cause of death from kittenhood up to 8 years old (45.58% of all cases). It is generally considered that older cats have a lower risk of mortality from trauma;^3,6^ therefore, we had expected RTA to be less prevalent in the older age group (aged 1–6 years). The risk factors previously identified for RTAs in cats aged less than 1 year were environmental, including living in a rural location, hunting at the roadside and living by a ‘long straight section of road’.^9^ Although environmental risk factors were not assessed in the current study, it is possible that for many cats their environment remained consistent, thus these risk factors persisted. As cats proceed into the mature adult life stage and beyond, behaviours that might increase the risk of RTAs, such as hunting and accessing the outdoors, may reduce as a result of diseases such as osteoarthritis and cognitive dysfunction. Older cats may also learn to avoid vehicles, or cats that have less ‘road sense’ may have succumbed to trauma earlier in life. Restricting cats of all ages to indoor environments can reduce the risk of RTAs by minimising exposure to this hazard. However, an indoor-only lifestyle may challenge reliably meeting all of a cat’s behavioural needs (eg, engagement in play, exploring, hunting, climbing and scratching), potentially inducing stress.^13,14^ While indoor-only living could effectively increase longevity, ensuring access to adequate environmental resources is essential to support normal feline behaviours.^13,15^

FIP represented the second most common cause of mortality in kittens and accounted for 3.87% of the deaths of all cats. These were not unexpected findings as it is well documented that FIP typically affects very young cats, with a second incidence peak in later life.^16,17^ At the time of data collection, FIP was still considered a fatal disease, with licensed antiviral drugs only becoming available in the UK in 2021. The success rate of treating FIP with antiviral medication has recently been reported to be in the range of 84.4–88.6%.^18^ It is therefore interesting to consider the impact antiviral treatment will have on all-cause mortality of kittens in the future.

In this study, 13.26% of all cases did not have a clearly attributable cause of death (non-specified category). This is likely multifactorial as a result of poor owner recollection, explained either by misunderstanding of veterinary terminology or reluctance to recall details from a traumatic event (death of a pet)^19^ as well as lack of definitive diagnosis if a cat was euthanased or died with vague presenting signs and/or without any diagnostic tests being performed.

Renal disease (both acute and chronic) was the third most common cause of death of all the cats in the study (6.63%), very closely followed by cardiovascular disease (6.35%), a pattern potentially influenced by the greater number of deaths in young adults compared with kittens. For cats aged 1–6 years, renal and cardiovascular disease both accounted for 6.52% of deaths, whereas 0% of kittens were affected by renal disease and 1.49% by cardiovascular disease. Renal disorders have previously been documented as the most common cause of mortality for cats aged 5 years and above in the UK;^2^ in Sweden, urinary causes of death (inclusive of renal disease) increased with age among an insured cohort.^6^ Although chronic kidney disease (CKD) is classically associated with older cats, there is no age-specific predilection for acute kidney injury (AKI) (eg, toxin exposure, pyelonephritis, ureteral obstruction, renal ischaemia, neoplasia and sepsis), and acute insults can lead to permanent chronic kidney dysfunction, which could explain the younger demographic represented in this study. Although typically associated with profound illness and marked clinical signs, patients with an AKI can also present with mild or non-specific clinical signs. Identification of persistent azotaemia therefore requires exclusion of acute processes (eg, by including urinary tract imaging and urinalysis) before a diagnosis of CKD can be reached. It has been suggested that CKD is under-diagnosed in younger cats; in a study of 100 randomly selected healthy cats in the USA, a high prevalence of CKD was found across three age groups, with a prevalence of 37.5% in cats aged 0–4.9 years, 40.9% in cats aged 5–9.9 years and 42.1% in cats aged 10–14.9 years, based on radiographic and biochemical assessment.^20^ If this reflects the general cat population, annual screening in younger life stages could potentially achieve earlier CKD diagnosis by tracking individual trends in renal biochemistry, thereby enabling proactive management (eg, optimally timed dietary interventions). In addition, knowledge of International Renal Interest Society stage 1 status should prompt judicious use of nephrotoxic drugs, vigilance against dehydration and blood pressure monitoring.^21^ These measures may slow disease progression, improve quality of life and potentially extend the cat’s lifespan.

For the purposes of this study, if a cat died from sudden death with a suggestion of cardiac disease documented in their medical history (eg, heart murmur, arrhythmia), their cause of mortality was classified as cardiovascular disease, since sudden death is a known clinical sign of feline cardiomyopathy. However, this is not specific; therefore, cardiovascular disease could have been over-represented by cases of sudden death. Conversely, mortality studies that rely on veterinary surveillance data may under-represent sudden death as a cause (and therefore possibly cardiovascular disease) as cats that die suddenly at home may not be presented to a veterinarian. The incidence of cardiovascular-related deaths in the present study increased with age, which likely parallels the increased prevalence of heart murmurs and dysrhythmias in older cats.^22^

The annual life expectancy of cats can vary significantly based on factors including age, sex, neuter status, breed and geographical location.^2,4–8^ Teng et al^5^ provided the first life tables for the UK pet cat population using surveillance data of cats from primary care clinics and gave an updated life expectancy of 11.74 years. The median longevity of cats in the UK had previously been reported as 13.5–14 years.^2,4^ Mortality risk factors were also assessed by Teng et al;^5^ being female, neutered and crossbred were associated with longer survival, whereas cats with body weights deviating significantly from the breed- and sex-specific median had shorter lifespans. Overall life expectancy could not be assessed in the present study owing to the use of data from an ongoing cohort study with cats not yet in their senior years. Specific risk factors, such as sex or breed, were not analysed because of small subgroup sizes owing to low mortality rates, but could be explored as the cohort ages further. The current study documented that the probability of death progressively rose with age after 2–3 years, in agreement with Teng et al,^5^ who reported the probability of death increasing from the age of 3–4 years onwards.

The limitations of this study include reliance on owner understanding and recollection, and therefore data are subject to reporting bias; in addition, medical records were not always available for corroboration. The overall sample size was small compared with to other similar studies. RTA accounted for nearly 50% of the all-cause mortality analysis for cats aged 8 years or less; therefore, all other categories contained only a small number of cases. Many cases were classified as non-specified, meaning some of the mortality causes may be under-represented. Categorisation of mortality causes was based on a presumptive diagnosis in all cases: none of the cats had a post-mortem examination. The sudden death category could be over-represented owing to lack of recognition or reporting of a prior illness by the owner. The age at death was approximate in some cases; this meant that all-cause mortality could not be assessed per year of age. Some cases had their age of death estimated to the nearest year, which could have resulted in under- or over-estimations of the age groups (by 1–11 months); however, these cases were excluded from the life table analysis and should not have affected the survival data. Data regarding the regularity of veterinary visits over the cat’s lifetime were not available at the time the cats died; thus, the proportion of deceased cats that had recently attended veterinary practices could not be determined.

Although the analysis attempted to account for dropouts, it cannot be discounted that some may be unreported deaths or that some owners may rejoin the study in the future. As owners were not always consistent in completing every questionnaire, the number of cats potentially incorrectly counted as dropouts was minimised by excluding the final two questionnaires from the analysis but considering their completion in the classification of dropouts; that is, owners would have had to have missed a minimum of the two most recent questionnaires to be classified as a dropout. This also allowed for lag in the reporting of cat deaths, to improve the accuracy of these numbers. However, the assumption that dropouts do not include mortality means the mortality figures are likely to be minimum numbers. To counteract missing data on mortality and true dropouts would require a hierarchical statistical analysis. This analysis would need to account for parameter uncertainty, and model death years and dropout rates as latent variables by drawing inference on age-specific mortality from confirmed deaths; however, the application of these analytical methods to account for such limitations is beyond the scope of this study. Future research could explore how a hierarchical modelling approach can be used to draw inference on age-specific survival when some of the records have missing information on timing of birth and death. In addition, this approach would then provide a more robust framework to evaluate the effect of both continuous and categorical covariates on survival.

## Conclusions

This study revealed that while kittens are more likely to succumb to age-related infectious diseases, trauma and accidents, organ-specific issues such as renal and cardiovascular diseases emerge as significant contributors to mortality in cats aged up to 8 years. RTAs prove to be the leading cause of death across all age groups studied. Mortality rates gradually increase from age interval 2–3 years, with a peak in mortality rate before this, and survival probabilities linearly decrease as cats age. An increased awareness of renal and cardiovascular disease in young adult cats should be considered, as earlier intervention could delay or reduce mortality. This study offers foundational knowledge about feline mortality that could be used to develop superior veterinary care strategies and thereby improve the longevity and quality of life for pet cats.
